# Editorial: CardioPulmonary Physiology: Novel Approaches to Pulmonary Function and Critical Care

**DOI:** 10.3389/fphys.2021.825098

**Published:** 2022-01-03

**Authors:** Zhanqi Zhao, Ling Sang, Tong In Oh

**Affiliations:** ^1^Department of Biomedical Engineering, Fourth Military Medical University, Xi'an, China; ^2^Institute of Technical Medicine, Furtwangen University, Villingen-Schwenningen, Germany; ^3^State Key Lab of Respiratory Diseases, Guangzhou Laboratory, Department of Critical Care Medicine, Guangzhou Institute of Respiratory Health, Guangzhou Medical University, The First Affiliated Hospital of Guangzhou Medical University, Guangzhou, China; ^4^College of Medicine, Kyung Hee University, Seoul, South Korea

**Keywords:** cardiopulmonary system, pulmonary function, critical care, electrical impedance tomography, early mobilization, chronic obstructive pulmonary disease, mechanical ventilation, lung perfusion images

The cardiopulmonary system is complex and highly regulated with interactions among lung ventilation, gas exchange, and pulmonary circulation determining respiratory outcomes. Cardiopulmonary physiology helps us integrate the cellular, multicellular, organ, and physiology of the respiratory system. Patients suffering from respiratory diseases, such as asthma, chronic obstructive pulmonary disease and idiopathic pulmonary fibrosis, require a range of pulmonary function tests to understand disease severity, including regular spirometry, diffusing capacity for carbon monoxide, forced oscillation technique for in depth mechanics etc. In cases of disease exacerbation or respiratory failure, patients may require ventilatory support in the intensive care unit. However, limited measures are currently available at the bedside to speculate on the status of the respiratory system (e.g., lung mechanics measured by ventilator, blood gasses). Novel measurement techniques that are laboratory-based, such as ultrasound for ventilation, electrical impedance tomography (EIT) and orthogonal polarization microscopy, show promise with respect to clinical practice, despite some failures. With novel developments in technologies and measurement modalities, our understanding in respiratory system grows rapidly. In this Research Topic, we were able to collect a series of research and reviews to deepen our understanding on the topic.

Scoliosis is deformity of the vertebral column. It causes displacement of intrathoracic organs, impaired rib movement and respiratory muscles contraction. Severe scoliosis decreases lung function and results in asymmetric lung function (Newton et al., 2005). Given the complexity of orthopaedic surgery, halo traction could be an option for preoperative patients. A halo is applied to a patient to allow traction using patients own weight. It helps to loosen up the spine and reduce curve magnitude for a safer surgery. Yang et al. provided a systematic review and meta-analysis to analyze the effect of halo traction on deformity and pulmonary function in severe scoliosis. They further confirmed that halo traction can improve pulmonary function, mainly in forced vital capacity (FVC) and Forced expiratory volume at 1 s (FEV1) %predicted, via reducing the degree of deformity in this patient group.

Halo traction is an invasive therapy. But exercise as simple as guided breathing, sitting up from supine, lifting arms may also help to improve lung functions for critically ill patients in the ICU (Schweickert et al., 2009). In this Research Topic, Eimer et al. investigate the effect of early mobilization on regional lung physiology, end-expiratory lung volume (EELV) and ventilation distribution with EIT. They found that ventilation and EELV in dorsal regions improved during mobilization but only the improvement of EELV remained after returning to initial position. In their study EIT was used to monitor the ventilation changes over time at the bedside. This novel imaging technology allows real-time non-invasive monitoring of ventilation distribution (Zhao et al., 2020). Two recent studies applying EIT to monitor lung aeration before, during and after physiotherapy showed similar findings that physiotherapy improved ventilation distribution (Longhini et al., 2020; Yuan et al., 2021). Together we have a better understanding why early physiotherapy can improve lung functions.

Patients with chronic obstructive pulmonary disease (COPD) have significant loss of lung functions. Smoking is a well-known cause of COPD. Not only active smokers but also passive smokers are the victims of cigarettes. In this collection, two related studies were presented to understand the role of ferroptosis in bronchoalveolar epithelial cell (BAEC) injury and the feasibility of early screening of pulmonary dysfunction. Lian et al. presented an experiment where BAEC was cultured in cigarette smoke extract. They found that cigarette smoke extract altered ferroptosis-related gene expression patterns of BAEC, which partially explained the underlying molecular mechanisms of cigarette smoke related lung injury. Frerichs et al. demonstrated that ventilation inhomogeneity in COPD occurs not only during FVC maneuver but also during normal tidal breathing. This finding indicated that EIT has the potential to identify pulmonary dysfunction without extreme cooperation of the COPD patients.

In the process of disease development, e.g., COPD exacerbation or respiratory failure, mechanical ventilation is a life-saving support. Unfortunately, inadequate settings of ventilator would further damage the respiratory system. To minimize ventilator-induced lung injury, lung protective ventilation strategies are implemented, including low tidal volume (The Ards Network, 2000) and adequate positive end-expiratory pressure (Hsu et al., 2021). However, the optimizations of ventilation mode and patient-ventilator interaction are less clear. Ge et al. demonstrated the first time in a randomized-controlled setting that airway pressure release ventilation was associated with better lung mechanics and hemodynamics compared to conventional ventilation mode in post cardiac surgery patients. Lin et al. revealed that reverse trigger also occurs in patients without acute respiratory distress syndrome. These findings provided important hints to reconsider our decision on ventilation mode and the need to monitor patient-ventilator asynchrony.

Besides the above-mentioned studies, we also have two in-depth reviews in this Research Topic. Bedside evaluation of lung perfusion with conductivity contrast EIT using saline bolus is a new clinical practice (He et al., 2020). Xu et al. introduced the implementation of the technique and summarized the experimental and clinical studies. They provided an overview of the status and progress of the technique and pointed out the direction of future studies. Lai and Huang summarized the mechanisms of pulmonary vascular endothelial hyperpermeability caused by mechanical ventilation or cyclic stretch. They delivered a comprehensive understanding of the research progress related to endothelial permeability.

We are excited to see so many submissions covering a wide range. Translations from knowledge of cardiopulmonary physiology to lung function measurement and patient care strategies are essential in clinical practice. We hope that this Research Topic may provide a firm standpoint for further studies.

## Author Contributions

ZZ drafted the manuscript. LS and TO critically revised the manuscript. All authors have approved the final version.

## Conflict of Interest

The authors declare that the research was conducted in the absence of any commercial or financial relationships that could be construed as a potential conflict of interest.

## Publisher's Note

All claims expressed in this article are solely those of the authors and do not necessarily represent those of their affiliated organizations, or those of the publisher, the editors and the reviewers. Any product that may be evaluated in this article, or claim that may be made by its manufacturer, is not guaranteed or endorsed by the publisher.
